# Anti-demineralization effect of desensitizer containing copolymer and sodium fluoride on root dentin – a transverse microradiographic study

**DOI:** 10.1080/23337931.2019.1591967

**Published:** 2019-03-20

**Authors:** Kazuaki Kawamura, Yuichi Kunimatsu, Takafumi Nakano, Haruhiko Hasegawa, Hirohisa Arakawa, Yoshiharu Mukai

**Affiliations:** a Division of Oral Health, Department of Oral Science, Graduate School of Dentistry, Kanagawa Dental University, Yokosuka, Japan;; b Division of Restorative Dentistry, Department of Oral Interdisciplinary Medicine, Graduate School of Dentistry, Kanagawa Dental University, Yokosuka, Japan

**Keywords:** Fluoride, copolymer, desensitizer, demineralization

## Abstract

**Objective:** To evaluate anti-demineralization effects of dentin desensitizer containing sodium fluoride and methacrylate-co-p-styrene sulfonic acid (MS polymer) on root dentin using transverse microradiography (TMR).

**Material and methods:** Twenty-four dentin specimens were divided into four groups: MSO (no fluoride), MSF (3000 ppm F), FJL (9000 ppm F), and Control. In MSO and MSF, each desensitizer was rubbed into the dentin surfaces for 10 s then left for 20 s. In FJL, paste containing 9000 ppm F was applied onto the surface for 30 s. All specimens, including the Controls, were rinsed with deionized water, dried and an area of their surface exposed to pH 5.0 acidic solution, refreshed every 24 h, for 4 days. Sections 300-µm-thick were assessed by TMR. Mineral profiles and integrated mineral loss (IML) of lesions were analyzed by dedicated software. IML was analyzed with one-way ANOVA and Tukey’s test.

**Results:** MSF and FJL specimens showed high mineral volume % at the surface and in lesions, and significantly lower IML than the other groups (*p* < .05).

**Conclusion:** Dentin desensitizer containing 3000 ppm fluoride and MS polymer has the same anti-demineralization effect as does a fluoride paste containing 9000 ppm F.

## Introduction

Dentin hypersensitivity is a common clinical condition typically manifested as a transient pain extending from the cervix to the root surface when stimulated by thermal, mechanical or chemical stimulation by for example cold water, brushing and fruit juice. It can be treated by occluding the dentin tubules with micro-crystals [1–3] or a bonding resin [4–6]. For example, MS (methacrylate-co-p-styrene sulfonic acid) polymer seals dentin tubules with calcium oxalate crystals [7]. Dentin hyper sensitivity might be induced by an excessive and inappropriate style of teeth brushing. People who develop hypersensitive teeth often do not clean them sufficiently due to pain from brushing. This increases the risk of caries of exposed root surfaces, which in turn exacerbates the hypersensitivity. Therefore, it is better to take preventive measures not only against the hypersensitivity but also against root caries.

It is widely known that fluoride treatment is effective at preventing root caries, and several treatments have been developed [8–11] and applied clinically. The first report that fluoride treatment is effective in improving the acid resistance of dentin was by Volker in 1939 [12]. Volker reported that treating dentin powder with sodium fluoride reduced the amount dissolved in acid. Since then, numerous similar reports have been made. Terenaka [13] reported that mouth wash with sodium fluoride at 100 ppm F is effective for preventing root caries. Gluzman [14] recommended applying silver diamine fluoride as professional care for primary prevention of root caries, and recommended using amorphous calcium phosphate dentifrice and 250 ppm NaF mouthwash for self-care. Nyvad [15] reported that in clinical trials 24 active root caries all became inactive by 2–6 months after being swabbed with 2% NaF (9000 ppm F) solution for 2 min at the start of the trial, and 2 months later in addition to normal daily brushing. Also, Sudjalim [16] reported 9000 ppm F tooth paste effectively prevented demineralization around orthodontic brackets. Calcium fluoride (CaF_2_) precipitates wherever the hard dental tissues are exposed to high concentrations of ionic fluoride, and acts as a fluoride reservoir [17], releasing fluoride over a long period due to its slow dissolution.

MS Coat F comprises MS polymer containing sodium fluoride, added to increase its acid resistance, at a concentration of 3000 ppm F [7]. These effects on bovine dental blocks have been studied using micro-computed tomography (micro-CT) [18]. In the current study, we compared the anti-demineralization effects of MS Coat F and Fluoro Jelly, which has a fluoride concentration as high as 9000 ppm, on dentin. The coated surface of bovine tooth was quantitatively evaluated using transverse microradiography (TMR). This evaluation technique, in which precise levels of mineral loss are shown, is widely recognized as the gold standard method in demineralization and remineralization studies of both enamel and dentin [19–21]. We hypothesized that the anti-demineralization effect of dentin desensitizer containing 3000 ppm fluoride and MS polymer would be same as that of a fluoride paste containing 9000 ppm F.

## Materials and methods

### Sample preparation and treatment

Twelve bovine incisors were extracted and the periodontal ligaments and other soft tissues removed. Their crowns were separated at the cementum-enamel junction (CEJ). Their roots were horizontally sectioned about 5 mm below the CEJ (Isomet Low Speed Saw, Buehler, IL, USA), then vertically sectioned into two halves. A flat experimental surface was made by cutting the root surface with a diamond-coated wire-sectioning machine (Well type 3242; Walter Ebner, Mannheim, Germany) 3 mm away from the pulp chamber. The surface of the dentin specimens was polished with 2000-grade water resistant paper (Fuji star DCCS, Sankyo Rikagaku, Saitama, Japan), washed with deionized water and cleaned ultrasonically (US-2R US Cleaner, AS ONE, Osaka, Japan) in deionized water at 10 °C for 5 min. A total of 24 specimens were randomly divided into four groups of six specimens as described later. Three specimens in each group were then fixed to the bottom of a plastic containers, thus each group had two containers. The specimens’ entire surface, except for a 2 × 3 mm area to serve as an experimental surface, was painted with acid-resistant varnish. The containers were then randomly sorted into four groups; MS Coat One (MSO) (Sun Medical, Okayama, Japan), MS Coat F (MSF) (Sun Medical, Okayama, Japan), Fluor Jelly (FJL) (Bee-Brand Medico Dental, Osaka, Japan) and the Control (Table 1). For the MSO and MSF groups, MS Coat One or MS Coat F desensitizer was rubbed into the dentin surfaces for 10 s and left in place for a further 20 s as per the manufacturer’s instructions. For the FJL group, Fluor Jelly was pasted onto the dentin surface for 30 s. After each treatment, specimens were rinsed with deionized water for 10 s and then dried with compressed air.

**Table 1.** Materials used.

Product name	Composition	pH	Fluoride conc.	Code	Manufacturer
MS Coat One	methyl methacrylate-co-p-styrene sulfonic acid (MS polymer) 1% oxalic acid water	1.5	0 ppm F	MSO	Sun medical
MS Coat F	methyl methacrylate-co-p-styrene sulfonic acid (MS polymer) 1% oxalic acid sodium fluoride water	2.0	3,000 ppm F	MSF	Sun medical
Fluoro jelly	sodium fluoride carmellose sodium water	3.5	9,000 ppm F	FJL	Bee brand medico dental

### Demineralization

The acid resistance test was performed by exposing the test surface to pH 5.0 acidic solution (1.5 mM Ca, 0.9 mM PO_4_, 50 mM acetic acid, 0.2 ppm F, pH 5.0) [22]. The amount of acidic solution per one specimen was 10 mL, therefore one container contained 30 mL of acidic solution. The solution was refreshed every 24 h. After 4 days, the specimens were rinsed with deionized water for 10 s and then dried with compressed air, and then immediately cut into 300-µm-thick sections and assessed by TMR as described below.

### Transverse microradiography（TMR）

The specimens were removed from the containers and gently washed with deionized water. From each specimen, a 300-µm-thick section was cut perpendicularly to the surface using a diamond-coated wire sectioning machine. The sections were placed on a Perspex holder in a droplet of water and covered with thin polyester sheets to avoid dentin shrinkage as previously described [23]. Together with an aluminum step wedge of 13 steps, ranging from 0 to 300 μm in thickness, the sections were radiographed on a high-resolution glass film plate (High-Resolution Plate, Konica-Minolta, Tokyo, Japan) with a nickel-filtered Cu-Kα source operated at 15 mA and 35 kV for 15 min (PW3830, Spectris, UK) . The radiographic images of the sections and aluminum step wedge were analyzed and the mineral content profiles of the lesions and IML values were measured by a microscope/video camera/microcomputer set-up and dedicated software (TMR2006 and 2012 Inspektor Research System, Amsterdam, Netherlands) [24,25].

*Statistical analysis –* One-way analysis of variance (ANOVA) and the Tukey test (SPSS-PC software version 21.0, IBM, Tokyo, Japan) were used to compare IML among groups. The level of significance was set at *p* < .05 level.

## Results

**Figure 1.**
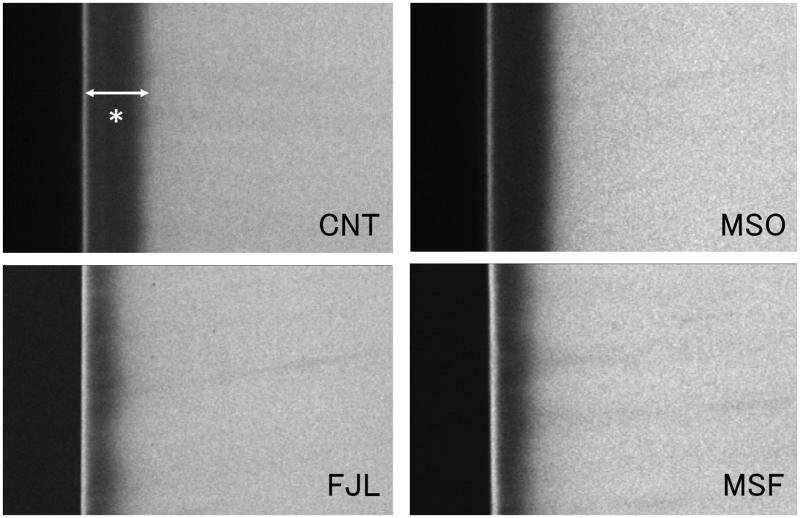
Representative TMR images. MSF and FJL had narrower demineralization zones than those of MSO and CNT. Also, MSF and FJL showed distinct radio-opaque surface layers as compared with other two groups.

**Figure 2.**
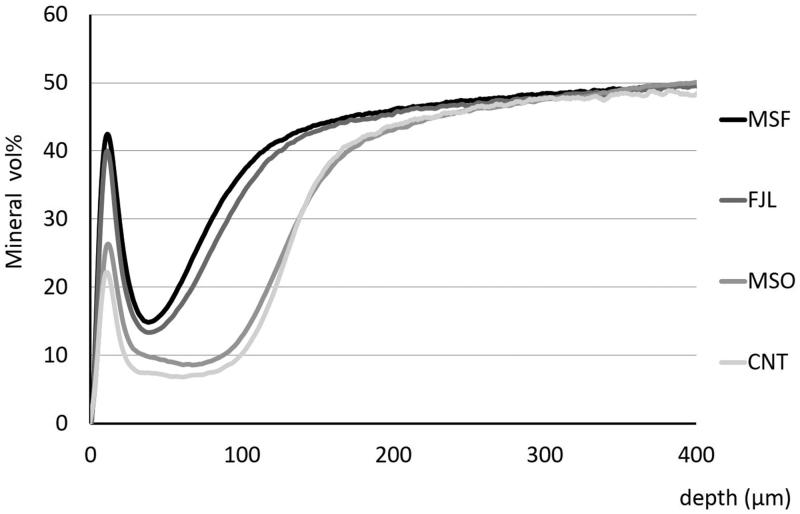
Average mineral profiles. MSF and FJL showed high mineral volume % at the surface and in lesion bodies compared to those of MSO and CNT groups.

Figure 1 shows representative TMR images of each group. MSF and FJL had narrower demineralization zones than those of MSO and CNT. Also, MSF and FJL showed distinct radio-opaque surface layers as compared with other two groups. In mean mineral profiles, MSF and FJL showed high mineral volume % at the surface and in lesion bodies compared to those of the MSO and CNT groups (Figure 2). The IML (vol % × µm) was 5698.0 ± 585.1 for CNT, 5457.3 ± 680.9 for MSO, 2853.8 ± 325.5 for MSF, and 3326.3 ± 461.4 for FJL, the values for MSF and FJL being significantly lower than those of the other two groups (*p* < .05) (Table 2).

**Table 2.** Integrated mineral loss of each group.

## Discussion

Root surface caries has become an important dental problem, especially in geriatric populations [26,27]. Carious lesions advance chronically and circularly at the gingival margin with extremely unclear borders, causing dentists much difficulty. In the current study, the effectiveness of a fluoride paste named Fluor Jelly, containing 9000 ppm F was compared with that of MS Coat F which contains 3000 ppm F. They were found to suppress root dentin demineralization to the same extent. MS Coat F is a desensitizing agent which contains MS polymer, 1% oxalic acid and sodium fluoride. The nano-sized MS polymer and oxalic acid both react with calcium on dentin. At the same time, fluoride ions are taken up into the dentin, and calcium oxalate, calcium fluoride and the MS polymer form a complex, which was anticipated to not only block dentin tubules but suppress demineralization at the root surface [7]. Although the fluoride concentration in MS Coat F is only a third of that in Fluor Jelly, it is thought that calcium fluoride and the MS polymer complex act as not only a mechanical protective film [28] but a fluoride ion releasing reservoir. Intraoral fluoride levels between 0.04 and 0.1 ppm may be found in saliva and plaque fluid when a fluoride toothpaste or rinse is used [29,30]. Fluoride levels in the sub ppm range were reported to shift the de-remineralization balance significantly *in vitro* [31], although higher levels of fluoride were needed to attain a similar degree of caries prevention when compared with enamel [32]. As a result, it is thought that effective anti-demineralization was obtained by a constant concentration of fluoride being supplied from MS Coat F to the tooth surface even while immersed in demineralizing solution, in which sub ppm fluoride concentration might be achieved. The 9000 ppm F Fluor Jelly may form CaF_2_ on the tooth surface, but the amount as a fluoride releasing reservoir may be insufficient because the material was applied for only 30 s, the same time as for the MS Coat F. That is, it was confirmed that MS Coat F exhibits the same demineralization inhibiting effect as does the fluoride coating agent with a fluoride concentration three times, even in the case of a short treatment time.

In some cases, hypersensitive dentin surfaces show no cavities or deficit. In such cases, application of dental filling materials or bonding systems may provide an environment prone to plaque accumulation. It is inadequate to restore these areas with filling materials as a first-choice treatment; they should preferably be covered with a thin layer. It was reported that MS Coat F produced a 2–3 µm-thick layer-like structure on the dentin surface [18].

It was reported that bovine root dentin can be used instead of human to evaluate caries development and inhibition [33]. Human teeth are subjected to various fluoride containing substances, such as toothpaste. To ensure the teeth compared did not have different histories of such exposure, bovine teeth were used in this study.

From the above, when MS Coat F is applied to the dental cervix, which is prone to accumulation of plaque and thus demineralization, fluoride released gradually from the material may inhibit demineralization beneath the dentin surface. Moreover, when combined with both phosphate and calcium ions from saliva or from fluid within the dentin tubules, it promotes deposition of fluoroapatite and other such minerals, which may contribute to long-term blockage of the tubules. Therefore, MS Coat F might be a medicament with caries prevention and durable desensitizing effects.

This study had some limitations. First, the effects were investigated in bovine teeth rather than human teeth for reasons discussed above. It is assumed the effects will generalize to human teeth but use of non-human teeth may be viewed as a limitation. Second, the anti-demineralizing effect of MSF was equal to that of 9000 ppm F paste, however, we did not use 3000 ppm F paste in this study. Some may point the possibility that fluoride at a concentration of 3000 ppm is sufficient and 9000 may be unnecessary for additional protection. An additional future study will be needed to clarify this point.

## Conclusion

In this *in vitro* study, the following conclusion could be drawn. A dentin desensitizer comprising MS polymer containing 3000 ppm fluoride had the same anti-demineralization effect as did a fluoride paste containing 9000 ppm F.

## Disclosure statement

No potential conflict of interest was reported by the authors.
